# Hypothalamic Subependymal Niche: A Novel Site of the Adult Neurogenesis

**DOI:** 10.1007/s10571-014-0058-5

**Published:** 2014-04-18

**Authors:** Ewa Rojczyk-Gołębiewska, Artur Pałasz, Ryszard Wiaderkiewicz

**Affiliations:** Department of Histology, Medical University of Silesia, ul. Medyków 18, 40-752 Katowice, Poland

**Keywords:** Adult neurogenesis, Hypothalamus, Stem cell niche, Energy balance

## Abstract

The discovery of undifferentiated, actively proliferating neural stem cells (NSCs) in the mature brain opened a brand new chapter in the contemporary neuroscience. Adult neurogenesis appears to occur in specific brain regions (including hypothalamus) throughout vertebrates’ life, being considered an important player in the processes of memory, learning, and neural plasticity. In the adult mammalian brain, NSCs are located mainly in the subgranular zone (SGZ) of the hippocampal dentate gyrus and in the subventricular zone (SVZ) of the lateral ventricle ependymal wall. Besides these classical regions, hypothalamic neurogenesis occurring mainly along and beneath the third ventricle wall seems to be especially well documented. Neurogenic zones in SGZ, SVZ, and in the hypothalamus share some particular common features like similar cellular cytoarchitecture, vascularization pattern, and extracellular matrix properties. Hypothalamic neurogenic niche is formed mainly by four special types of radial glia-like tanycytes. They are characterized by distinct expression of some neural progenitor and stem cell markers. Moreover, there are numerous suggestions that newborn hypothalamic neurons have a significant ability to integrate into the local neural pathways and to play important physiological roles, especially in the energy balance regulation. Newly formed neurons in the hypothalamus can synthesize and release food intake regulating neuropeptides and they are sensitive to the leptin. On the other hand, high-fat diet positively influences hypothalamic neurogenesis in rodents. The nature of this intriguing new site of adult neurogenesis is still so far poorly studied and requires further investigations.

## Introduction

Over the last hundred years, neuroscience has approved a presumption that the process of neurogenesis is not present in the structures of the adult brain. Beginning with the pioneer studies by Santiago Ramon y Cajal and Camillo Golgi, it was commonly thought that neural cells forming mature CNS are definitively postmitotic, do not arise de novo, and that plasticity adaptation mechanisms are based only on the origin of projections and synaptic connections between neurons formed in early brain development stages (de Castro et al. 2007). Mitotic divisions of ependymal cells located along lateral ventricles of mature rat brain were found for the first time by Ezra Allen already in 1912. However, only after recognition of molecular mechanisms of DNA synthesis, it was possible to unequivocally identify undifferentiated, proliferatively active neural stem cells (NSCs) in mature brain (Allen 1912; Balu and Lucki 2009). It was achieved in 1965 by Joseph Altman group (Altman and Das 1965) using tritium labeled thymidine to detect replicating DNA of precursor cells. This discovery opened the brand new, promising, and intriguing chapter of contemporary neurobiology. Nevertheless, the significance of the adult neurogenesis is still far from being explained. A number of recent studies unequivocally reveal the role of this phenomenon in the processes of CNS plasticity, learning, and memory (Deng et al. 2010; Yang et al. 2010; Monteiro et al. 2014; Ikrar et al. 2013).

The problem of relations between adult neurogenesis and pathogenesis or/and course of psychiatric diseases like bipolar disorder (Walton et al. 2012; Braun and Jessberger 2014), schizophrenia (Reif et al. 2007; Eisch et al. 2008; Christian et al. 2010; DeCarolis and Eisch 2010; de Koning et al. 2013), anxiety disorders (Kheirbek et al. 2012), and particularly depression (Krishnan and Nestler 2008; Lucassen et al. 2010; Mahar et al. 2014) is currently broadly discussed. Experiments on animal models have proven that elevated levels of stress hormones, observed in depression, often correlated with neurogenesis process reduction. Numerous antidepressants, commonly used in the therapy of patients suffering from depression, have strongly stimulating influence on new neurons formation in particular brain structures (Santarelli et al. 2003; Boldrini et al. 2009). However, there are some doubts and assumptions stating that attenuation of neurogenesis is not a cause of depression, but it is rather one of its functional outcomes (Airan et al. 2007). For quite a long-time postulated association between disturbances in formation, proliferation, and differentiation of NSCs and pathogenesis of neurological diseases, Alzheimer’s (Veereraghavalu et al. 2010; Sun et al. 2009; Rodriguez et al. 2008) and Parkinson’s (Bertilsson et al. 2008; Arias-Carrion et al. 2007) also seems to be of great importance. It is suggested that decrease of self-renewal rate of certain neural cells populations, e.g., in substantia nigra, hippocampus, or some neocortex areas **(**Bonfanti and Peretto 2011 **),** could constitute one of the causes of mentioned diseases. Based on this assumption, intensive search for the substance selectively stimulating neurogenesis in CNS is currently in progress. Relatively promising and quite well-studied aminopropyl carbasole and its newly synthesized derivatives (Pieper et al. 2010; Tesla et al. 2012; De Jesús-Cortés et al. 2012; Yoon et al. 2013) might become potential, but still hypothetical drugs being able to improve clinical state of patients with neurodegenerative disorders. A decrease in proliferation rate and survival of mature brain neural progenitors is probably related to chronic stress (Mirescu and Gould 2006) and long-lasting insomnia (Meerlo et al. 2009) as well. On the other hand, some data suggest cautiously that among consequences of ischemic stroke and traumatic brain injury, there is also stimulation of adult neurogenesis as one of potential brain repair mechanisms (Kernie and Parent 2010; Bellenchi et al. 2013; Braun and Jessberger 2014).

## Adult Neurogenenesis in the Canonical Sites

**Fig. 1:**
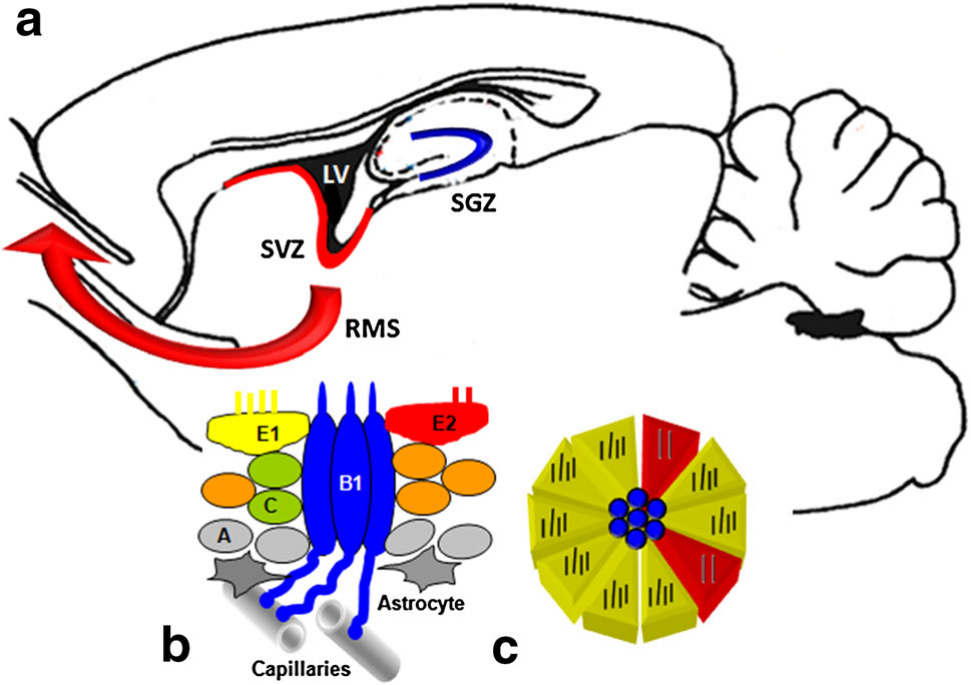
The canonical sites of adult neurogenesis in the rat brain and the scheme of subventricular zone (SVZ) stem cell niche. **a** Subgranular zone of dentate gyrus (SGZ, *dark blue*), subventricular zone (SVZ, *red*) surrounding the lateral ventricle (LV, *black*), *red arrow* indicates direction of neuron migration from SVZ to olfactory bulb (rostral migratory stream, RMS). **b** Cellular composition of SVZ niche sectioned vertically to ependymal surface; E1 cells (*yellow*) and E2 cells (*red*) concentrically surround centrally located cilia of B1 cells (*dark blue*) forming the “pinwheel” structure. Neuroblasts—A cells (*gray*) and transiently activated stem cells C (*green*) are also presented. **c** Horizontal view of the mentioned SVZ niche (Color figure online)

Up till now, two distinctive regions of active and constant neurogenesis process in adult mammalian CNS have been identified: subgranular zone (SGZ) in dentate gyrus structure and subventricular zone (SVZ) located subependymally in the vicinity of brain lateral ventricles (Fig. 1). SGZ stem cells are the source of dentate gyrus granular cells, whereas SVZ forms progenitor cells migrating as rostral migratory stream (RMS) to olfactory bulb, where they differentiate into interneurons suitable for this brain area. The neural precursor cells (NPCs) must be located in neurogenic niche, a permissive milieu, which maintain their constant ability to divide and form mature neurons even in the adult brain. The hippocampal SGZ niche is created mainly by astrocytes and blood vessel endothelium, numerously represented in this layer of dentate gyrus (Rolando and Taylor 2014). In SGZ cytoarchitectonics three types of proliferatively active cells have been identified: (1) radial glia-like stem cells, type I cells; (2) non-ciliated cells with nestin expression, type II cells also described as transiently activated progenitor cells, TAP cells; and (3) neuroblasts with doublecortin protein (DCX) and Ki67 expression. On the other hand, in SVZ structure, four mitotically active and self-renewing cell populations have been distinguished: (1) ependymocytes with CD133 expression; (2) GFAP-positive astrocytic stem cells, type B1 cells; (3) transiently activated progenitor (TAP) cells with Mash1 expression, type C cells; and (4) DCX protein expressing neuroblasts, type A cells (Okano and Sawamoto 2008).

Ependymocytes play a fundamental role in SVZ niche structure formation. Here, three cell subpopulations have been distinguished: previously mentioned CD133^+^ cells functioning as NSCs as well as E1 and E2 ependymal cells with CD24 expression. Multiciliated E1 cells are dominating, whereas E2 ependymocytes make up less than 5 % of ependymal cell population and possess only two cilia with particularly big basal bodies. Studies of rat brain SVZ niche cytoarchitecture have revealed unusually peculiar spatial pattern of stem cells and ependymocytes. Central part of the niche is formed by GFAP-positive B1 stem cells surrounded in their apical, ciliated part by several E1 and E2 ependymocytes forming a unique rosette or pinwheel-like structure (Mirzadeh et al. 2008). On the other hand, central and basal part of B1 cells remain in contact with concentrically located C and A cells. B1 cells have long cytoplasmatic projections reaching the surface of blood capillaries (Fig. 1).

Extracellular matrix, in the form of three-dimensional spatial structures called fractones, plays also an important role in the niche functioning. In human and rodent brain, fractones are characterized by collagen IV, β1 and γ1 laminin, perlecan and nidogen expression, and lacking laminin β2 (Kerever et al. 2007). Limited fractone subpopulation surrounding highly mitotically active cells additionally demonstrates immunoreactivity toward *N*-sulfate heparan sulfate proteoglycan (*N*-sulfate HSPG). Fractones and subependymal capillaries are probably capable of binding fibroblast growth factor 2 (FGF-2), which substantially promotes NSCs proliferation (Kerever et al. 2007). Matrix metaloproteinases—MMP-1 and MMP9—are also considered to play a significant role in internal niche environment modification and NSCs fate determination (Tonti et al. 2009).

The origin of neurons in the SGZ and SVZ is probably strictly regulated by various growth factors (Duan et al. 2008; Balu and Lucki 2009; Llorens-Martín et al. 2009; Pałasz et al. 2010), classical neurotransmitters (Petrus et al. 2009; O’Keeffe et al. 2009; Yanpallewar et al. 2010), neuropeptides (Howell et al. 2005; Garza et al. 2008; Moon et al. 2009; Manda and Reiter 2010), and cytoskeleton proteins (Jiao et al. 2008; Sibbe et al. 2009; Kunze et al. 2009; Gascon et al. 2010). A large spectrum of commonly administered pharmaceuticals such as selective serotonin reuptake inhibitors (Encinas et al. 2006; Jaako et al. 2009), antipsychotics (Wang et al. 2004; Keilhoff et al. 2010; Nandra and Agius 2012; Benninghoff et al. 2013), hypnotic and normothymic drugs (Yu et al. 2009; Boku et al. 2010), lithium ions (Wexler et al. 2008) and even steroid hormones (Brummelte and Galea 2010), and cyclooxygenase-2 inhibitors (Goncalves et al. 2010) can also significantly affect the course of adult neurogenesis.

There are numerous evidences, that new neural cells can also be formed, usually in limited number, outside SGZ and SVZ, in potential niches located in distinct structures of mature brain. Their presence has been proven in hypothalamus (Kokoeva et al. 2005; Xu et al. 2005; Migaud et al. 2010), amygdala (Bernier et al. 2002; Luzzati et al. 2003), striatum (Emsley et al. 2005; Bédard et al. 2006), and substantia nigra (Zhao and Janson Lang 2009). Multipotent neural precursors have been also isolated from certain neocortical areas (Dayer et al. 2005; Vaysse et al. 2012) of adult brain. Currently, there is not enough evidence proving that these putative, non-classical neurogenic sites are stable in time and space, and that the continuous origin of new neurons is really taking place. Therefore, the presence of established and permanently functional NSCs niches in mentioned brain areas is still very controversial. However, hypothesis suggesting existence of a stable hypothalamic neurogenic site located in subependymal zone of the third ventricle (hypothalamic ventricular zone, HVZ) seems to be particularly intriguing and relatively well documented (Kokoeva et al. 2005; Yuan and Arias-Carrion 2011; Cheng 2013). More importantly, it is also suggested that neurogenesis process is significantly involved in various hypothalamic regulatory mechanisms, especially in the energy balance regulation (Pierce and Xu 2010).

## Structure of Hypothalmic Neural Stem Cell Niche

**Fig. 2:**
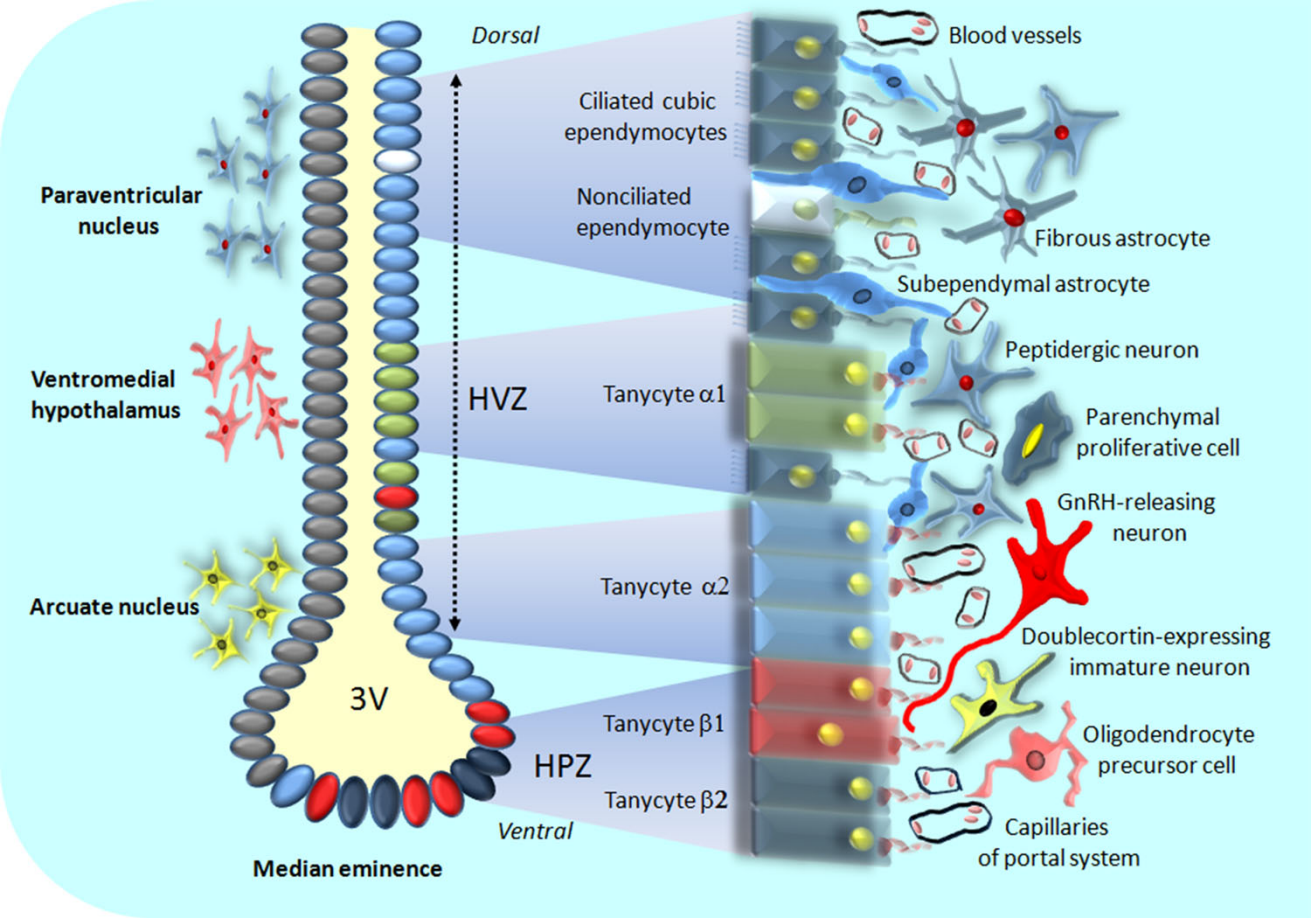
Schematic representation of the subependymal NPCs niche in the animal hypothalamus. The neurogenic zones are located in the vicinity of the third ventricle at the level of paraventricular, ventromedial, and arcuate nuclei (hypothalamic ventricular zone, HVZ), while tanycytes of the median eminence form the hypothalamic proliferating zone (HPZ). Ciliated and non-ciliated ependymocytes line the third ventricle as well as four types of tanycytes. Various populations of glial cells (fibrous astrocytes, subependymal astrocytes, and oligodendrocyte precursor cells) and neural cells (peptidergic neurons, parenchymal proliferative cells, GnRH-releasing neurons, and doublecortin-expressing immature neurons) are located beneath the ependymal layer forming the NPCs niche. Neuropeptides and other regulatory factors are released from the capillary network

The key role of ependymocytes lining lateral ventricles has become a source of presumptions that these cells could also take part in hypothalamic NSCs niche formation, located in the vicinity of the third ventricle. Studies held on rodents have revealed that proliferatively active cells are present not only in ependyma, but also in surrounding neuropilus (Kokoeva et al. 2007; Migaud et al. 2010). To date, two adult hypothalamic neurogenesis regions have been distinguished: located in lateral walls of the third ventricle at the level of paraventricular and arcuate nuclei (hypothalamic ventricular zone, HVZ) and hypothalamic proliferating zone (HPZ) formed by tanycytes located at the bottom of the third ventricle in median eminence region (Fig. 2). Tanycytes that form rat and human HVZ are characterized by expression of proteins typical for neural precursor cells e.g., nestin (Wei et al. 2002), vimentin (Bolborea and Dale 2013 ), and doublecortin-like protein (DCL) (Saaltink et al. 2012). They are also enriched for neural stem and progenitor genes such as Sox9, Notch 1 and 2, Hes 1 and 5, CD63, FZD5, Dirc, NTrk-2T1, and Thrsp (Rodríguez et al. 2005; Shimogori et al. 2010; Lee and Blackshaw 2012). Recently, the expression of Sox2, a selective marker of NSCs, was also reported (Lee et al. 2012; Li et al. 2012). Four types of radial glia-like tanycytes have been identified, differing from each other in gene profile and location in the third ventricle wall. Tanycytes α1 are present at the level of ventromedial nuclei, whereas α2 subpopulation in the vicinity of arcuate nuclei. Both cell types send their long processes toward blood vessels, hypothalamic neurons, and glial cells. Elongated β1 tanycytes form the lateral part of infundibular recess, while β2 cells line the floor of third ventricle inside the median eminence forming the HPZ. The basal processes of β1 tanycytes are in contact with endothelial cells and terminals of GnRH-expressing neurons, whereas processes of β2 cells head toward blood vessels of hypothalamo-pituitary portal system and also pia mater (Lee and Blackshaw 2012) (Fig. 2). Noteworthy, the β2 tanycytes show particularly high level of aforementioned Hes1 and Hes2 marker expression (Lee et al. 2012). Some authors report the presence of the following zones of the third ventricle wall: ventrally located tanycytic zone composed of both β and α2 tanycytes, transition zone with α1 tanycytes, and the most dorsal ependymal zone which contains only ependymal cells (Mathew 2008). Most recently, two kinds of tanycytes have been found, dorsally located FgF10−, BLBP−, GFAP+, GLAST+, S100β+ α subtype and ventral FgF10+, BLBP+, GFAP−, GLAST− β subtype (Haan et al. 2013). Probably, the Fgf10+ tanycytes are the neural stem/progenitor cells and can continuously proliferate and form parenchymal neurons and glial cells. Importantly, tanycytes located on the median eminence are sensitive to the hormones and nutritional substances carried by the blood flowing in capillary vessels. In turn, the somata of tanycytes lining the third ventricle are able to receive the molecular signals e.g., growth factors from the cerebrospinal fluid.

The identity of hypothalamic neural progenitor cells (NPCs) remains so far very controversial. Some studies suggest that β-tanycytes are the main proliferating cells in the hypothalamus of young adult mice (Haan et al. 2013). They are the neural progenitors in the median eminence but not in other hypothalamic regions. In rats, the rate of neurogenesis within HVZ and median eminence is significantly higher in the first weeks of postnatal life than in adult animals (Lee et al. 2012). On the other hand, a recent report identifies the lateral α-tanycytes as local NPCs and crucial cells in the mouse hypothalamic niche. The long-term study using a lineage tracing in vivo shows that α-tanycytes form a self-renewing population that differentiate to new tanycytes, astrocytes, and neurons. Moreover, this finding demonstrates clearly that only GFAP-positive dorsal α-tanycytes, but not β-tanycytes or parenchymal cells may form neurospheres (Robins et al. 2013). Probably, the fibroblast growth factor (FGF) signaling is required to maintain α-tanycyte proliferation, and increased FGF level enhances the rate of mitotic divisions. Interestingly enough, the hypothalamic tanycytes are characterized by selective expression of transcription factor called retina and anterior pituitary neural fold homeobox (Rax). This molecule shows a similar pattern of expression to retinoic acid receptor responder (Rarres-2), a marker of third ventricle ependymal cells. The Rax action is considered as required for tanycyte and ependymocyte differentiation at the level of hypothalamus (Miranda-Angulo et al. 2014). The regulatory mechanism by which Rax determines the fate of tanycytes is still unknown; however, some recent studies suggest the involvement of Six6 and Hmgb2, the factors selectively expressed in hypothalamic progenitor cells (Andreazzoli et al. 2003, Shimogori et al. 2010). Acetylcholine, histamine, and ATP sensitive tanycytes can activate intracellular calcium signaling regulatory pathway (Frayling et al. 2011). It should be noted that ATP stimulates Ca^2+^ waves in the subventricular progenitor cells acting via P2Y1 receptor (Weissman et al. 2004; Pearson et al. 2005). Tanycytes as the radial glial cells can release ATP to control neural progenitor cell proliferation, and they also have the aforementioned type of purynergic receptors. Moreover, tanycytes synthesize ectonucleoside triphosphate diphosphohydrolase 2 (NTPDase 2) that inactivate the extracellular ATP (Braun et al. 2003).

**Fig. 3:**
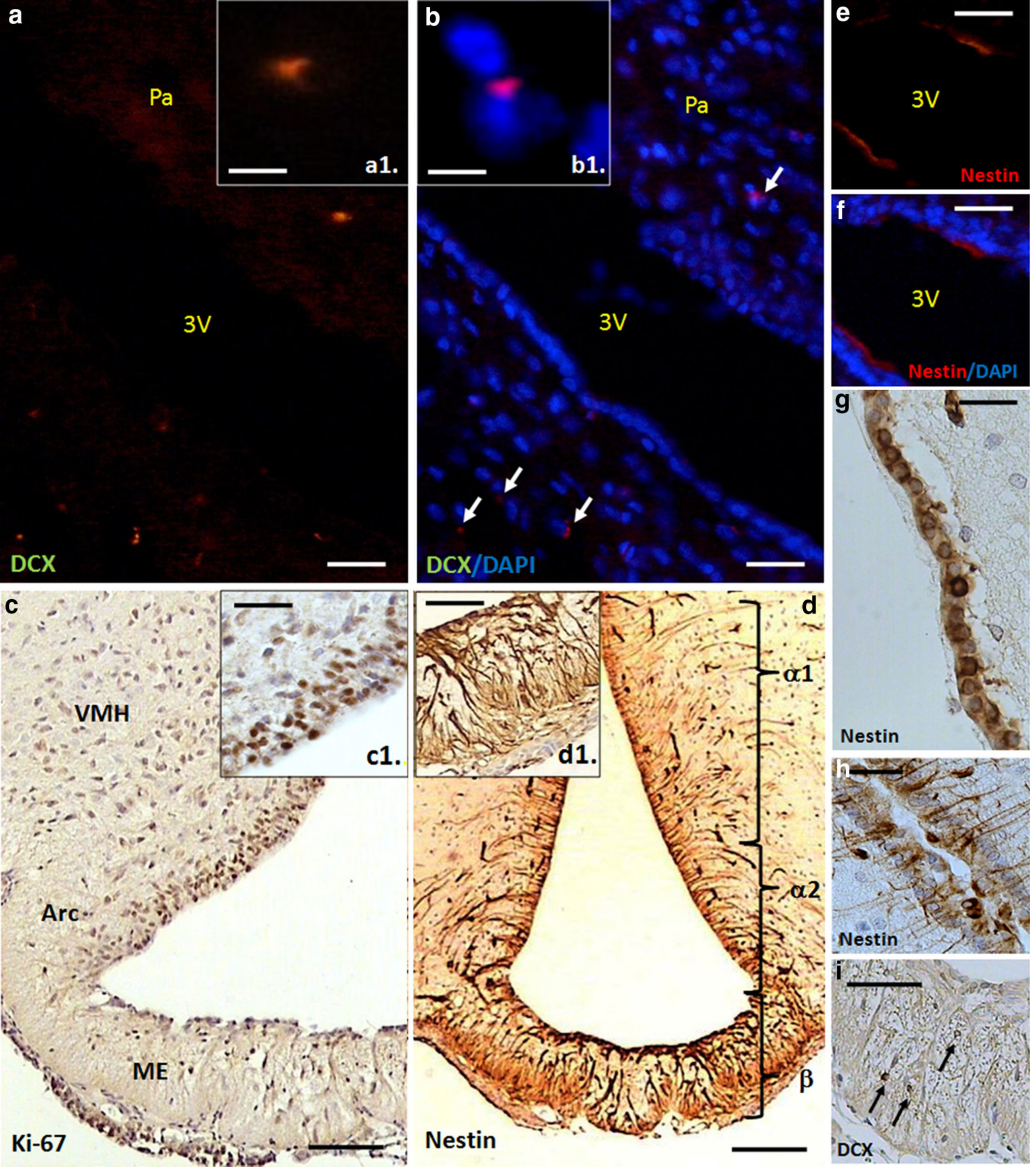
Hypothalamic neurogenic zones in the rat brain—fluorescence (20-μm-thick frozen sections) and classical immunostaining (7-μm paraffin sections) of the adult (3-month-old) rat hypothalamus. Primary antibodies dilutions: DCX 1:100 (Santa Cruz Biotechnology), nestin 1:500 (Millipore), and Ki67 1:100 (Abcam). Brains were fixed with 4 % buffered formalin, dehydrated with ethanol or sucrose solutions, and then embedded on paraffin or mounted in OCT medium. The photomicrograph shows the doublecortin (DCX) expressing cells in the parenchyma of paraventricular nucleus (**a**, **b**, a single cell in higher magnification—*insets* a1, b1) and in the median eminence (**i**). Ki-67-positive dividing cells in the subependymal zone (**c**, **a** tanycyte layer—*inset* c1). Nestin expressing cells: tanycytes at the level of ventral hypothalamus, arcuate nuclei and within the median eminence (**d**, zone of β tanycytes—*inset* d1, α1 tanycytes, **h**), and ependymocytes in the dorsal HVZ (**e**–**g**). *Scale bars* 50 μm, excluding **c**, **d**, **i** (100 μm), and a1, b1 (10 μm). *Arc* arcuate nucleus, *ME* median eminence, *Pa* paraventricular nucleus, *VMH* ventromedial hypothalamus, *3V* third ventricle

Although tanycytes appear to be the key players in the NPCs niche formation, they are not only NSCs in the hypothalamus. A population of SOX2 expressing cells scattered within the parenchymal regions also has a proliferative activity (Kokoeva et al. 2005; Li et al. 2012). Undoubtedly, advanced genetic tools such as using of inducible Cre lines e.g., hGFAPCreER^T2^, PDGFRα-CreRT^T2^, or GLAST-CreER^T2^ should be useful to reveal whether these BrdU-positive cells can form a quiescent hypothalamic neural progenitor pool (Ganat et al. 2006; Rivers et al. 2008; de Melo et al. 2012; Lee and Blackshaw 2012). The proliferating GFAP-positive subependymal astrocytes have an apical process with single cilium and their periventricular basement membranes form a three-dimensional network that is typical for SVZ microachitecture (Pérez-Martin et al. 2010). Numerous granular DCX-positive immature neuroblasts were identified close to the wall of the third ventricle (Fig. 3). On the other hand, the bipolar DCX cells found in the parenchyma of ventromedial hypothalamus have fusiform perikarya and sometimes elongated processes displaying the features of migrating cells that may undergo maturation within this region (Batailler et al. 2013). Perhaps, the neuroblasts located inside the subependymal niche can form a kind of migratory stream spreading to the hypothalamic nuclei (Haan et al. 2013).

A large spectrum of endogenous regulatory substances, especially growth factors, is able to regulate the course of adult hypothalamic neurogenesis. The brain-derived growth factor (BDNF), insulin-like growth factor 1 (IGF-1), fibroblast growth factor (FGF), epidermal growth factor (EGF), and ciliary neurotrophic factor (CNTF) seem to play a key role as the activators of neuronal progenitor divisions. A recent report suggests that also gonadotropin-releasing hormone (GnRH) can increase the proliferating activity of hypothalamic NPCs in aged mice (Zhang et al. 2013). It should be emphasized that the aforementioned growth factors promote neurogenesis in different hypothalamic centers in a distinctly selective manner (Sousa-Ferreira et al. 2014). Thus, BDNF administration increases cell proliferation in the paraventricular nucleus, both FGF and CNTF induce neurogenesis in the parenchymal zone of the arcuate nucleus, while IGF-1 in the medial periventricular region (Pencea et al. 2001; Pierce and Xu 2010; Pérez-Martin et al. 2010). Interestingly, the single simultaneous i.c.v. injection of FGF and EGF promotes cell proliferation in the ependymal layer, whereas extended subcutaneous injection increases neurogenesis in the arcuate nucleus (Xu et al. 2005; Rivera et al. 2009).

## Functional Aspects of Hypothalamic Neurogenesis

As hypothalamus is responsible for the constant control of many autonomous functions of the organism, it was considered that neurogenesis in this region may support some of these processes or act as a compensatory mechanism in response to different environmental signals. Indeed, in the light of latest discoveries, it is possible that neurogenesis in hypothalamus is potentially involved in regulation of energy homeostasis via modulation of eating behavior.

**Fig. 4:**
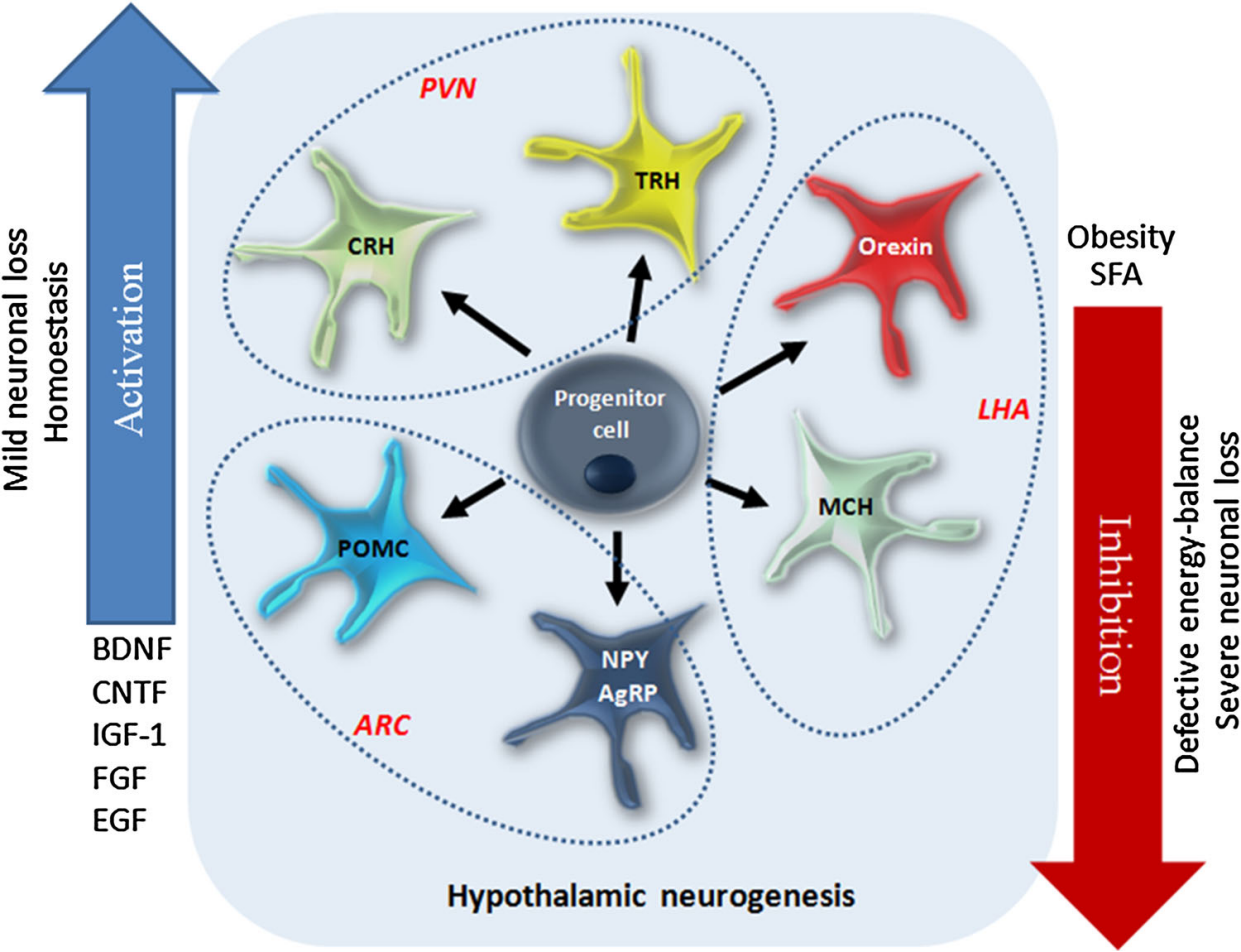
Hypothalamic neurogenesis plays a role in the regulation of energy balance. The limited neuronal loss, homeostasis, and some growth factors promote the NPCs proliferation, while severe cellular loss, obesity, and saturated fatty acids (SFA) inhibit the neurogenesis. *ARC* arcuate nucleus, *PVN* paraventricular nucleus, *LHA* lateral hypothalamus, *POMC* pro-opiomelanocortin, *NPY* neuropeptide Y, *AgRP* agouti-related protein, *CRH* corticotrophin-releasing hormone, *TRH* thyrotropin-releasing hormone, *MCH* melanin-concentrating hormone, *BDNF* brain-derived neurotrophic factor, *CNTF* ciliary neurotrophic factor, *IGF-1* insulin-like growth factor 1, *FGF* fibroblast growth factor, *EGF* epidermal growth factor

Neuronal populations located within distinct regions of the hypothalamus express various neuropeptides modulating food intake. In the arcuate nucleus (Arc), there is an expression of orexigenic factors like agouti-related peptide (AgRP), neuropeptide Y (NPY), as well as anorexigenic factors like pro-opiomelanocortin (POMC) and its derivative—α-melanocyte-stimulating hormone (α-MSH). On the other hand, latheral hypothalamus (LHA) expresses two peptides promoting food intake—orexin and melanin-concentrating hormone (MCH) (Fig. 4) (Sousa-Ferreira et al. 2014). In normal conditions, there is a dynamic balance between regulatory neuropeptides activities, but diet-related factors like obesity and high saturated fatty acid (SFA) intake cause hypothalamic neurogenesis inhibition and simultaneous defective energy balance. It suggests a link between eating behavior and intensity of hypothalamic neurogenesis (Fig. 4) (Sousa-Ferreira et al. 2014).

It was also shown that newborn neural cells in the hypothalamus are functional—they express orexigenic peptides (AgRP, NPY)—and anorexigenic peptide (POMC) (Kokoeva et al. 2005; Pierce and Xu 2010). Simultaneously, some of them have ability to respond to leptin administration by inducing strong phospho-signaling transducer and activator of transcription 3 (pSTAT3) immunoreactivity (Kokoeva et al. 2005). Interesting data concerning significance of hypothalamic neurogenesis have been provided with the use of antimitotic agent arabinosylcytosine (AraC) or focal irradiation procedure—both applied in order to silence neurogenesis process. In wild-type mice, ablation of AgRP-expressing neurons leads to the weight loss. However, this effect is not observed in mutant mice with degenerated AgRP-positive cells. As such genetic manipulation causes also the increase in cell proliferation rate in arcuate nucleus of the hypothalamus and because inhibition of this proliferation (by AraC) results in decreased feeding and weight loss, it is suggested that hypothalamic neurogenesis plays a compensatory role in preventing anorectic effects of neurodegeneration (Pierce and Xu 2010). In another study, mice were kept on high-fat diet (HFD) and infused with CTNF to induce neurogenesis within hypothalamic energy balance centers (Kokoeva et al. 2005). CTNF treatment resulted in weight loss, but the long-term effect was present only in mice with undistrupted neurogenesis process. If AraC was administered, then mice gained weight 20 days after CTNF treatment, which indicates that neurogenesis in hypothalamus is crucial for maintaining CTNF-mediated metabolism regulation (Kokoeva et al. 2005; Migaud et al. 2010). Later, it was found that HFD alone enhances neurogenesis in the median eminence (ME) of mouse hypothalamus. Blocking of this process by focal irradiation leads to attenuation of weight gain and increase in animal activity levels, which proves that neurogenesis in ME may promote fat storage and body mass increase (Lee et al. 2012; Lee and Blackshaw 2012). On the other hand, studies on rodent obesity models revealed the significant decrease in hypothalamic neurogenesis process rate in arcuate nucleus of obese mice. It further suggests that regulation of energetic homeostasis can be mediated by hypothalamic neurogenesis (McNay et al. 2012; Sousa-Ferreira et al. 2014).

As tanycytes of the median eminence are considered as new neural cells precursors, detailed studies have been performed to characterize these cells and trace the fate of their progeny. Experiments held on mice confirmed that tanycytes positive for fibroblast growth factor 10 (FGF10^+^) give rise to cells that are located mainly in arcuate nucleus. Some of them expressed NPY and were sensitive to fasting alone or followed by leptin administration, which was proven by c-fos and pSTAT immunostaining. Moreover, newly formed neurons were able to significantly expand their axonal/dendritic arborization (Haan et al. 2013). Tanycytes are also known to respond to glucose by releasing ATP Ca^2+^ waves, especially if it is applied selectively to cell bodies (Frayling et al. 2011). Another interesting feature of tanycytes, suggesting their contribution to energy metabolism, is expression of orphan receptor GPR50—member of melatonin receptor family (Sidibe et al. 2010). GPR50 is functionally linked to energy homeostasis, which was proven by observation of knock-out (GPR50^−^) mice having more stable body weight—they were more resistant to obesity induced by high-energy diet and to fasting-induced weight loss (Ivanova et al. 2007; Bolborea and Dale 2013). Another intriguing point of view, from which adult hypothalamic neurogenesis can be analyzed, is the relationship between this process, obesity, and macronutrients dietary content. Diet rich in saturated fatty acids (SFA) not only induced obesity and pre-diabetes in mouse model, but also caused reduction in the number of NSCs and their proliferation potential, which was associated with inhibitor kappaβ/nuclear factor kappaB (IKKβ/NFκB) activation (Li et al. 2012; Yon et al. 2013). Different effects were observed when rats were fed with SFA-rich diet during pregnancy. Resulting offspring showed higher proliferation rate of NPCs together with the increase in blood circulating lipids and orexigenic peptides expression (Chang et al. 2008; Yon et al. 2013). Additionally, some researchers linked neurogenesis process with endocannabinoid system. Treatment with the cannabinoid-1 receptor inverse agonist (AM251) reduced food intake and modulated neurogenesis in rats, but the neurogenic change was present only in HFD-fed animals—there was a reduction of neurogenesis levels in SVZ and hypothalamus, but induction in SGZ. It suggests that HFD sensitizes the endocannabinoid system to modulate neurogenesis (Rivera et al. 2011; Yon et al. 2013).

Apart from well-documented contribution of neurogenesis in hypothalamus to energy balance regulation, some other potential functions of newly formed neural cells have been reported (Yuan and Arias-Carrion 2011). It is supposed that social environment—especially pheromones—influences hypothalamic neurogenesis in adult female prairie voles. Two-day-long male exposure increased the number of new formed cells in amygdala and hypothalamus in comparison to condition of female exposure and social isolation (Fowler et al. 2002). Moreover, immunohistochemical staining of female pig hypothalamus have revealed that new oxytocin-containing neuron formation rate in paraventricular nucleus (PVN) is greater in lactating sow and adult gilts in comparison to puberty ones (Raymond et al. 2006). In another study held on female pigs, formation rate of new neurons expressing vasopressin in vasopressin and oxytocin-containing nucleus (VON) was positively correlated with the growth of this brain region—more newly formed vasopressin positive neurons were shown in adult pigs, than in adolescent individuals (Rankin et al. 2003). It may support the hypothesis that adult hypothalamic neurogenesis is involved in sexual maturation process and can be sensitive to environmental conditions. The pubertal increase in anteroventral periventricular nucleus (AVPV) volume seems to be connected with the hormone-dependent formation of new cells (Juraska et al. 2013). In the study by Ahmed et al. (2008), BrdU was administered to rats that were at different stages of puberty, and the labeled cell was quantified 3 weeks later. A number of AVPV cells that had been born during puberty had transformed into mature neurons. Interestingly, more BrdU cells were found in the female than in the male AVPV. Unexpectedly enough, BrdU-labeled cells were also found in the sexually dimorphic nucleus of the preoptic area (SDN), with greater numbers of pubertally born cells in males than in females (Ahmed et al. 2008). However, these BrdU-positive cells coexpressed neither neural nor glial markers, so their phenotype remains unclear. Possibly, they require more than 3–4 weeks to become the fully differentiated neurons or astrocytes, as suggested for cortical interneurons (Cameron and Dayer 2008; Juraska et al. 2013).

## Conclusions

Recently, accumulating reports seem to confirm the real existence of adult hypothalamic neurogenesis in the animal brain and highlight its potential role in the energy balance regulation. The cellular architecture of hypothalamic subependymal neurogenic niche is quite well described; however, many crucial questions remain so far unanswered. First of all, the presence of NPCs in the human hypothalamus is not yet reliably proven. Moreover, we also do not know whether hypothalamic neurogenesis can contribute to energy homeostasis in humans. Both eating disorders and obesity pathogenesis are strictly related to the disturbances at the level of hypothalamic neuronal populations. The blockage of hypothalamic neurogenesis may alter the body weight in rodents supporting the hypothesis that local NPCs play role in the control of energy expenditure.

Having in mind that hypothalamic neurogenesis may be regulated by various factors, an intriguing idea of potential pharmacomodulation of this process should not be excluded. It would be interesting to study the influence of some drugs modulating appetite (as their main or side effect) on hypothalamic neurogenesis process in order to better understand their mechanism of action. In the future, it could have some practical implication allowing modification and improvement of existing therapies interfering with organism energetic homeostasis—for example, treatment of eating disorders and schizophrenia.

Taking to the account recent results from experiments on pigs and prairie voles, it is also justified to perform research on hormonal regulation of hypothalamic neurogenesis and its impact on various physiological processes connected with hormonal activity. Finally, promising future direction of research concerns hypothalamus-derived NSCs cultures. It would contribute to detailed characterization of neural precursors and resulting application of hypothalamic regenerative potential.
